# Valor dos exames clínicos para o diagnóstico de rupturas do ligamento cruzado anterior: O que há de novo?

**DOI:** 10.1055/s-0045-1813006

**Published:** 2025-12-10

**Authors:** Son Van Truong, Uyen Thi Phuong Nguyen, Long Thanh Nguyen

**Affiliations:** 1 Departamento de Cirurgia, Danang Hospital, Danang, Vietnã; 2 Departamento de Radiologia, Vinhduc Hospital, Quangnam, Vietnã

**Keywords:** lesões do ligamento cruzado anterior, lesões do joelho, menisco, ruptura, anterior cruciate ligament injuries, knee injuries, meniscus, rupture

## Abstract

**Objetivo:**

Investigar o valor dos exames clínicos para diagnóstico de rupturas do ligamento cruzado anterior (LCA) com os testes da gaveta anterior, Lachman, *pivot-shift* , sinal de alavanca e sinal de flambagem ativa forçada (FAB, do inglês *forced active buckling* ).

**Métodos:**

Este estudo transversal foi conduzido com 165 pacientes com lesão no joelho e indicação para artroscopia, entre janeiro e dezembro de 2022. Os resultados dos exames clínicos foram comparados ao padrão-ouro do procedimento para determinar o valor dos testes diagnósticos.

**Resultados:**

Os valores de sensibilidade e especificidade foram, respectivamente: teste da gaveta anterior: 77,5% e 86,1%; Lachman: 87,6% e 88,9%; *pivot-shift* : 65,9% e 94,4%; sinal de alavanca: 93,8% e 94,4%; e sinal de FAB: 81,4% e 97,2%.

**Conclusão:**

Há diversos exames clínicos para rupturas do LCA. O sinal da alavanca é um teste clínico útil para exame e diagnóstico dessa condição.

## Abstract (en)

**Objective:**

To investigate the value of clinical diagnostic tests for diagnosing anterior cruciate ligament (ACL) tears with the anterior drawer, Lachman, Pivot-shift, Lever sign, and Forced Active Buckling (FAB)sign tests.

**Methods:**

A cross-sectional study was conducted on 165 knee injury patients who were indicated for knee arthroscopy from January to December 2022. The clinical examination results were compared with the procedure's gold standard to determine the value of clinical diagnostic tests.

**Results:**

The sensitivity and specificity values, respectively, were anterior drawer test: 77.5% and 86.1%; Lachman: 87.6% and 88.9%, Pivot-shift: 65.9% and 94.4%; Lever sign: 93.8% and 94.4%; and FAB sign: 81.4% and 97.2%.

**Conclusion:**

There are various clinical diagnostic tests for ACL tears. The Lever sign is a useful clinical test for physicians to examine and diagnose ACL tears.

## Introdução

O ligamento cruzado anterior (LCA) é fundamental para a estabilização da articulação do joelho, impedindo o deslocamento anterior excessivo para frente e a rotação interna da tíbia. Uma ruptura parcial ou completa do LCA constitui uma lesão substancial no joelho. Sua taxa de incidência anual na população geral varia de 30 a 80 casos por 100.000 pessoas-ano. Essa lesão é notavelmente prevalente entre indivíduos mais jovens, com cerca de 70% dos casos sendo decorrentes de traumas relacionados à prática de esportes. 1 2

O diagnóstico precoce de lesões do LCA é crucial para escolha do esquema terapêutico adequado e otimização dos desfechos. 3 O padrão-ouro para essa condição é a imagem artroscópica direta, mas os achados da ressonância magnética (RM) também são considerados um padrão de referência, com sensibilidade e especificidade entre 94 e 98%. 4 No entanto, nem todas as unidades de saúde em países menos desenvolvidos têm acesso aos equipamentos diagnósticos necessários. Assim, o exame clínico para a detecção de lesões do LCA continua sendo bastante valioso.

Os três métodos clínicos mais aceitos para o diagnóstico de rupturas do LCA são o gaveta anterior, Lachman e *pivot-shift* . Em 2014, o médico italiano Allesandro Lelli publicou um trabalho sobre o teste do sinal de alavanca (também conhecido como teste de Lelli), realizado em 400 pacientes. Em comparação à RM, os resultados preliminares mostraram uma sensibilidade de quase 100% no diagnóstico de rupturas parciais ou completas do LCA, tanto agudas quanto crônicas. Este é um método clinicamente viável que não depende da gravidade da lesão. 3

Em 2020, o cirurgião ortopédico alemão Fabian Blanke introduziu um novo método clínico para o diagnóstico de rupturas do LCA em fase subaguda, denominado sinal de flambagem ativa forçada (FAB, do inglês *forced active buckling* ). Acredita-se que este exame tenha maior sensibilidade e especificidade do que os testes de Lachman e *pivot-shift* . 5 Para aprimorar o arsenal diagnóstico de rupturas do LCA na prática clínica, a presente pesquisa foi conduzida para investigar o valor de diversos exames clínicos para a detecção de lesões do LCA.

## Materiais e Métodos

### Participantes

Este estudo transversal foi realizado em 165 pacientes com lesão no joelho e indicação para artroscopia no Hospital Danang entre janeiro de 2022 e dezembro de 2022. Os critérios de inclusão foram: indivíduos com (1) idade igual ou superior a 14 anos, (2) histórico de lesão no joelho e (3) cirurgia artroscópica unilateral agendada. Foram excluídos do estudo aqueles com histórico de cirurgia no joelho, infecção no joelho, doenças bilaterais do joelho ou fraturas adjacentes ao joelho.

Todos os participantes assinaram o termo de consentimento livre e esclarecido. O estudo foi conduzido de acordo com a Declaração de Helsinque e aprovado pelo comitê de ética sob o número HDYD2021/45, em 15 de dezembro de 2021.

### Delineamento Experimental

Os pacientes foram selecionados para o estudo com base nos critérios da agenda cirúrgica diária. Foram registradas informações gerais, como idade, sexo, lesão no joelho, causa da lesão, estágios da lesão (agudo < 3 semanas; subagudo 3–12 semanas; crônico, ≥ 12 semanas). Em seguida, os exames clínicos pré-operatórios foram avaliados por um único cirurgião ortopédico (sem conhecimento do diagnóstico do paciente).

As artroscopias de joelho foram realizadas por nosso cirurgião sênior, especializado em cirurgia do joelho, que desconhecia os resultados dos exames físicos. Os resultados intraoperatórios (presença ou ausência de ruptura do LCA) foram documentados. A seguir, os resultados dos exames clínicos foram comparados ao padrão-ouro para determinar o valor dos testes clínicos.

### Exames Físicos

Os testes de gaveta anterior, Lachman e *pivot-shift* foram conduzidos como descrito na literatura. 2

Para realização do teste do sinal de alavanca (ou teste de Lelli), o paciente ficou deitado em decúbito dorsal sobre uma superfície rígida com o joelho totalmente estendido. O examinador colocou uma mão sob o terço inferior da panturrilha e exerceu uma força moderada para baixo sobre o terço inferior do quadríceps com a outra mão. O teste é positivo quando o calcanhar não se levanta da mesa de exame. Esse resultado sugere uma ruptura do LCA, pois a tíbia não consegue se alavancar para frente com a força aplicada no fêmur. Por outro lado, o teste é negativo quando o calcanhar se levanta da mesa. 6

No sinal de FAB (para exame do membro inferior esquerdo), o paciente foi colocado em decúbito dorsal. O examinador se posicionou ao lado esquerdo do paciente e flexionou o quadril esquerdo até que o joelho estivesse em cerca de 20 a 30° de flexão. Com a mão esquerda, o examinador segurou o tornozelo do paciente e aplicou uma rotação interna à perna, enquanto mantinha a mão direita sobre o joelho, exercendo uma leve força de rotação externa. O paciente era, então, instruído a estender ativamente o joelho. O teste é positivo se houver subluxação tibial anterior, o que indica ruptura do LCA. O teste é negativo caso o paciente consiga estender o joelho por completo, sem subluxação tibial. 5

### Análise Estatística

A análise estatística foi realizada pelo IBM SPSS Statistics for Windows (IBM Corp.), versão 20.0. A sensibilidade, a especificidade, o valor preditivo positivo (VPP), o valor preditivo negativo (VPN) e a acurácia foram utilizados para descrever o desempenho diagnóstico.

## Resultados

A idade média dos 165 pacientes foi de 37,3 ± 12,4 anos (17–65 anos). No total, 107 pacientes eram do sexo masculino e 58 do feminino. As lesões acometeram 90 joelhos esquerdos e 75 joelhos direitos. As principais causas de lesão foram acidentes de trânsito (41,3%) e lesões relacionadas à prática de esportes (44,2%), como apresentado na Tabela 1 .

**Tabela 1:** Dados demográficos e epidemiológicos basais

Variáveis	Número (%)
**Idade, anos (média ± DP)**	37,3 ± 12,4 [17;65]
**Sexo (masculino/feminino)**	107 (64,5) / 58 (35,2)
**Lado (esquerdo/direito)**	90 (54,5) / 75 (45,5)
**Causas da lesão**	
Acidentes de trânsito	68 (41,3)
Lesões relacionadas à prática de esportes	73 (44,2)
Acidentes pessoais	18 (10,9)
Acidentes de trabalho	6 (3,6)
**Estágios da lesão**	
Agudo	54 (32,7)
Subagudo	61 (37,0)
Crônico	50 (30,3)
**Diagnóstico artroscópico (presença/ausência de lesão LCA)**	129 (78,2) / 36 (21,8)

**Abreviações:** LCA, ligamento cruzado anterior; DP, desvio padrão.

Os testes clínicos com alta sensibilidade no diagnóstico de rupturas do LCA foram o sinal da alavanca (93,8%), Lachman (87,6%) e o sinal de FAB (81,4%). Os com especificidade mais alta foram o sinal de FAB (97,2%), o sinal da alavanca (94,4%) e o *pivot-shift* (94,4%). Todos os 5 testes clínicos têm alto valor preditivo positivo, superior a 90% ( Tabela 2 ).

**Tabela 2:** Valores diagnósticos dos cinco testes para detecção de rupturas do LCA

Itens	GA	TL	TPS	TSA	FAB
Verdadeiro-positivo, n	100	113	85	121	105
Verdadeiro-negativo, n	31	32	34	34	35
Falso-positivo, n	5	4	2	2	1
Falso-negativo, n	29	16	44	8	24
Sensibilidade, %	77,5	87,6	65,9	93,8	81,4
Especificidade, %	86,1	88,9	94,4	94,4	97,2
Acurácia, %	79,4	87,9	72,1	93,9	84,8
VPP, %	95,2	96,6	97,7	98,4	99,1
VPN, %	51,7	66,7	43,6	81,0	59,3

**Abreviações:** FAB, sinal de flambagem ativa forçada (do inglês, *forced active buckling* ); GA, teste da gaveta anterior; LCA, ligamento cruzado anterior; TL, teste de Lachman; TPS, teste de *pivot-shift* ; TSA, teste do sinal de alavanca; VPP, valor preditivo positivo; VPN, valor preditivo negativo.

## Discussão

O LCA é uma estrutura crucial para a manutenção da estabilidade da articulação do joelho, tornando essencial o diagnóstico para o tratamento eficaz e a prevenção de outras lesões nesta articulação. Tradicionalmente, o diagnóstico clínico de rupturas do LCA baseia-se em três testes: gaveta anterior, Lachman e *pivot-shift* .

O teste da gaveta anterior é historicamente o método mais popular e utilizado. No entanto, não apresenta a sensibilidade necessária para diagnóstico de rupturas agudas do LCA em comparação a lesões crônicas. O teste de Lachman é considerado o método mais preciso e confiável para o diagnóstico de rupturas do LCA, enquanto o *pivot-shift* é o mais específico, porém menos sensível, dos três. 7 8

Em nosso estudo, observamos que a sensibilidade e a especificidade do teste da gaveta anterior foram de 77,5% e 86,1%, do Lachman 87,6% e 88,9% e do *pivot-shift* 65,9% e 94,4%. Na recente meta-análise de Huang et al., 9 a sensibilidade e a especificidade do teste da gaveta anterior foram de 64% e 87%, do Lachman de 76% e 89%, e do *pivot-shift* de 59% e 97%, respectivamente. Sokal et al. 10 constataram que a sensibilidade e a especificidade do teste da gaveta anterior foram de 83 e 85%, do Lachman de 81% e 85%, e do *pivot-shift* de 55% e 94%, respectivamente.

Nossos resultados são compatíveis aos relatados na literatura. Todos esses estudos indicaram que a acurácia dos testes clássicos é influenciada por vários fatores, como aumento de volume, inflamação articular reativa e proteção muscular devido à dor. 11 Além disso, a mão pequena do examinador ou o pé grande do paciente pode dificultar a realização desses testes e levar a resultados imprecisos. 12

Em nosso estudo, introduzimos dois testes clínicos recentemente desenvolvidos, o sinal de alavanca e o sinal de FAB, que apresentam diversas vantagens. Sua simplicidade, facilidade de administração e independência da experiência do examinador os tornam particularmente valiosos em cenários clínicos desafiadores. Os resultados mostraram que o sinal de FAB apresenta alta especificidade diagnóstica, de 97,2%, semelhante a do sinal de alavanca (94,4%) e do *pivot-shift* (94,4%). Por outro lado, o sinal de FAB apresenta sensibilidade superior, de 81,4%, comparado ao *pivot-shift* , de 65,9%.

Tanto o sinal de FAB quanto o teste de *pivot-shift* compartilham um mecanismo comum para a detecção de lesões do LCA, que é a subluxação da tíbia em relação ao côndilo femoral. O sinal de FAB, porém, é menos doloroso para o paciente *pivot-shift* , o que pode facilitar a obtenção de uma resposta positiva durante o exame. O sinal de FAB, desenvolvido por Blanke et al., 5 relatou sensibilidade e especificidade de 78 e 95%. As diferenças entre estes resultados e os nossos achados podem ser atribuídas ao estudo original incluir somente pacientes com lesões após 6 semanas ou mais, 5 enquanto não tivemos limitação de tempo dentre os pacientes selecionados.

O sinal da alavanca é o teste com maior sensibilidade e especificidade (93,8% e 94,4%, respectivamente). Os autores do teste do sinal da alavanca, Lelli et al., 6 relataram 100% de sensibilidade. No entanto, Kulwin et al. 13 mostraram que a sensibilidade e a especificidade do sinal da alavanca foram bastante baixas, de 44,4% e 88,3%, respectivamente, em comparação à RM.

Em meta-análises, o sinal da alavanca apresentou resultados relativamente favoráveis. Huang et al. 9 relataram uma sensibilidade de 79% e especificidade de 92%, enquanto no estudo de Sokal et al. 10 os valores foram 83% e 91%, respectivamente.

Na prática clínica, descobrimos que os resultados dos testes são influenciados pela mesa de exame. Caso o paciente seja posicionado em uma mesa acolchoada pode ocorrer um resultado falso-negativo. Portanto, conduzimos os testes em uma mesa de exame rígida ou colocamos uma tábua sob o pé do paciente para obter resultados mais precisos. Além disso, a escolha do posicionamento da mão sob a panturrilha é crucial durante o exame. Nossa experiência é examinar primeiro a perna saudável, escolher um ponto sob a panturrilha em que a elevação do calcanhar é mais alta e, então, usá-lo como referência para exame da perna lesionada.

Outro fator que pode afetar os resultados do sinal da alavanca é a presença de lesões condrais. Massey et al. 14 observaram que sua precisão diminui de 89 para 74% na presença de lesão condral, com significância estatística. Este fator pode explicar por que alguns estudos relatam que o sinal da alavanca apresenta sensibilidade relativamente baixa.

## Conclusão

Existem diversos testes de clínicos para o diagnóstico de rupturas do LCA. O método diagnóstico ideal deve ser de fácil realização e ter alta sensibilidade e especificidade. Neste contexto, o sinal da alavanca é um teste clínico útil para exame e diagnóstico dessa condição. Nossos resultados demonstraram que ele não apenas é um teste prático e simples, mas também apresenta acurácia diagnóstica superior em comparação aos métodos tradicionais. Isso o torna uma ferramenta inestimável, em especial em ambientes onde o acesso a técnicas avançadas de diagnóstico por imagem é limitado.
